# HIV-1 Induced Bystander Apoptosis

**DOI:** 10.3390/v4113020

**Published:** 2012-11-09

**Authors:** Himanshu Garg, Jonathon Mohl, Anjali Joshi

**Affiliations:** Center of Excellence for Infectious Disease, Department of Biomedical Science, Texas Tech University Health Sciences Center, 5001 El Paso Dr, MSB-1 Annex, El Paso, TX 79905, USA; Email: jon.mohl@ttuhsc.edu (J.M.); anjali.joshi@ttuhsc.edu (A.J.)

**Keywords:** HIV, AIDS, apoptosis, bystander, Env, CCR5, immune activation, fusion, hemifusion, gp41

## Abstract

Apoptosis of uninfected bystander cells is a key element of HIV pathogenesis and believed to be the driving force behind the selective depletion of CD4+ T cells leading to immunodeficiency. While several viral proteins have been implicated in this process the complex interaction between Env glycoprotein expressed on the surface of infected cells and the receptor and co-receptor expressing bystander cells has been proposed as a major mechanism. HIV-1 utilizes CD4 as the primary receptor for entry into cells; however, it is the viral co-receptor usage that greatly influences CD4 decline and progression to AIDS. This phenomenon is relatively simple for X4 viruses, which arise later during the course of the disease, are considered to be highly fusogenic, and cause a rapid CD4+ T cell decline. However, in contrast, R5 viruses in general have a greater transmissibility, are encountered early during the disease and have a lesser pathogenic potential than the former. The above generalization gets complicated in numerous situations where R5 viruses persist throughout the disease and are capable of causing a rigorous CD4+ T cell decline. This review will discuss the multiple factors that are reported to influence HIV induced bystander apoptosis and pathogenesis including Env glycoprotein phenotype, virus tropism, disease stage, co-receptor expression on CD4+ T cells, immune activation and therapies targeting the viral envelope.

## 1. Introduction

HIV infections cause a progressive depletion of a select group of immune cells namely the CD4+ T helper cells leading to immunodeficiency. While HIV directly and selectively infects CD4+ T cells, the low levels of infected cells in patients is discordant with the rate of CD4+ T cell decline and argues against the role of direct infection in CD4 loss. In agreement with this, in natural Simian Immunodeficiency Virus (SIV) infections in the wild there is no loss of CD4+ T cells or immunodeficiency in infected animals despite high levels of viremia [1,2]. Early in the 1990s, Gougeon *et al.* proposed that apoptosis was involved in the selective loss of CD4+ T cells via unknown mechanisms [3]. This led to a plethora of studies trying to determine the mechanism of apoptosis induction during HIV infection [4,5,6,7,8]. While it is now clear from both *in vitro* and *in vivo* studies as well as *ex vivo* culture of Peripheral Blood Mononuclear Cells (PBMCs) from HIV infected individuals that apoptosis is one of the major cause of CD4+ T cell loss in HIV infections, the mechanism behind this phenomenon still remains highly debated [9]. It has been proposed that CD4+ T cell loss may be attributed to one of the following (1) Direct cell killing due to infection; (2) Apoptosis induced by viral proteins like the Env, Tat, Nef, Vpu, Vpr (reviewed by Gougeon 2005) [10]; (3) Cell death due to excessive activation of immune cells-activation induced cell death [11]; (4) Bystander apoptosis of neighboring uninfected cells [9]. Of these, bystander apoptosis appears to encompass an explanation for most of the phenomenon observed during HIV infection that lead to progression to AIDS and remains one of the leading hypothesis for CD4+ T cell loss [3,12,13].

## 2. Bystander Apoptosis

It is evident from studies over the years that direct infection is not sufficient to account for all the CD4 loss in HIV infections. This has led to the belief that HIV is able to kill uninfected bystander cells via apoptosis [3]. The first direct evidence for this came from studies by Finkel *et al.* [6] who demonstrated in lymph node sections that the majority of cells undergoing apoptosis during HIV infection are not actually infected but lie in close proximity of infected cells. While the mechanism of bystander CD4+ T cell loss remains highly debated, the role of the Env glycoprotein in this process is becoming increasingly evident [9,14,15]. This is largely supported by the following arguments: (1) Cell death in HIV infection outnumbers the infected cell population, (2) Depletion of immune cells is restricted to CD4+ helper T cells and as the Env glycoprotein binds to CD4 it most likely plays a role, either directly or indirectly, in CD4+ T cell death. (3) The Env glycoprotein is expressed on the surface of infected cells and can interact with bystander cells expressing CD4 and a co-receptor CXCR4/CCR5. The interaction of Env with bystander cells via the receptor and co-receptor is relatively complex and involves a number of factors that can influence this phenomenon. The same factors that influence Env interaction with bystander cells are also likely to affect bystander apoptosis mediated by Env glycoprotein. Some of the key factors that regulate this process are Env glycoprotein phenotype, virus tropism, disease stage, co-receptor expression on CD4+ T cells, immune activation and therapies targeting the viral envelope.

## 3. Env Glycoprotein Mediated Fusion

The primary purpose of Env glycoprotein is to facilitate the fusion of viral and cellular membranes resulting in viral entry. The Env glycoprotein of HIV is arranged on the surface of the virus and virus-infected cells as a hetero-trimer. Each monomer is composed of a receptor-binding surface unit (gp120) and a fusogenic transmembrane unit (gp41) that mediates fusion of membranes [16,17]. The gp120 subunit binds to CD4 and a co-receptor either CXCR4 (X4) [18] or CCR5 (R5) [19] on T helper cells. Binding of HIV gp120 to CD4 triggers a complex sequence of events involving several conformational changes in gp120 that result in exposure of co-receptor binding sites on gp120 and the N-terminal and C-terminal heptad repeat (HR) regions of gp41. Subsequently gp41 HR domains interact with each other to form a six helix bundle [17]. Interaction of these domains in a leucine zipper like fashion allows effector and target membranes to come in close proximity resulting in fusion of target and viral membranes [20].

 However, recent studies suggest that the process of Env fusion is more complex than previously thought. It is now believed that the HIV Env not only facilitates infection of isolated cells but that productive transmission of virus occurs at the contact site between infected and uninfected cells referred to as the virological synapse [21]. This interaction across the virological synapse involves the active participation of various cellular and viral components including CD4, CXCR4, Env glycoprotein [22], adhesion molecules like Lymphocyte Function-Associated Antigen-1 (LFA-1), Intercellular Adhesion Molecules (ICAM-1 and ICAM-3) [23] tetraspanins, [24] as well as actin and tubulin cytoskeletal proteins [25]. Thus, the HIV Env glycoprotein has several functions, including but not limited to: (1) facilitating entry of viral nucleocapsid into the target cell thereby permitting virus replication (2) aiding in virus transmission across the virological synapse (3) playing a role in HIV pathogenesis by inducing apoptosis in uninfected bystander cells [26]. 

## 4. Env Glycoprotein Mediated Bystander Apoptosis

Although the role of the Env glycoprotein is primarily to mediate fusion of the viral and cellular membranes allowing for viral entry, it is also known that the HIV Env glycoprotein is capable of inducing CD4+ T cell apoptosis. Laurent-Crawford *et al.* [4] were the first to demonstrate that the HIV Env glycoprotein alone expressed on the surface of cells is capable of inducing cell death in neighboring T cells. Through the use of HIV Env expressing cells cocultured with uninfected CD4+ T cells, they provided first direct evidence for the role of viral Env in inducing apoptosis in T cells in the vicinity. Since then this coculture approach has become the standard for various groups studying the phenomenon of bystander apoptosis. 

That HIV Env binds CD4 and a co-receptor suggests that these cell surface expressed receptors play a pivotal role in HIV Env mediated bystander apoptosis. This is supported by the observations that inhibiting Env CD4 interactions also inhibits Env mediated apoptosis [7]. Subsequently, studies conducted with the CXCR4 antagonist AMD3100 demonstrated that inhibition of the Env co-receptor interactions also inhibits apoptosis [27]. This led to the idea that binding of Env to CD4 and CXCR4 receptor is critical for the process of apoptosis. In a study by Biard-Piechaczyk *et al*., the authors selectively inhibited signaling via CD4 and CXCR4 and found that the conventional signaling pathways known to be associated with these receptors are not involved in the process of Env mediated apoptosis [7]. Later, Blanco *et al*. demonstrated that inhibiting the Env mediated fusion process using gp41 fusion inhibitors abolishes bystander apoptosis [28]. This correlation between HIV fusogenic activity and bystander apoptosis has also been demonstrated by others [15,29,30]. While the process of fusion mediated by Env glycoprotein is complex, it is clear that it involves several sequential steps starting with gp120 binding to CD4 and a co-receptor and culminating in gp41 mediated membrane fusion. Hence, inhibiting this process at a later stage in the fusion process suggests that gp41 function is critical for the phenomenon of apoptosis. 

**Figure 1:**
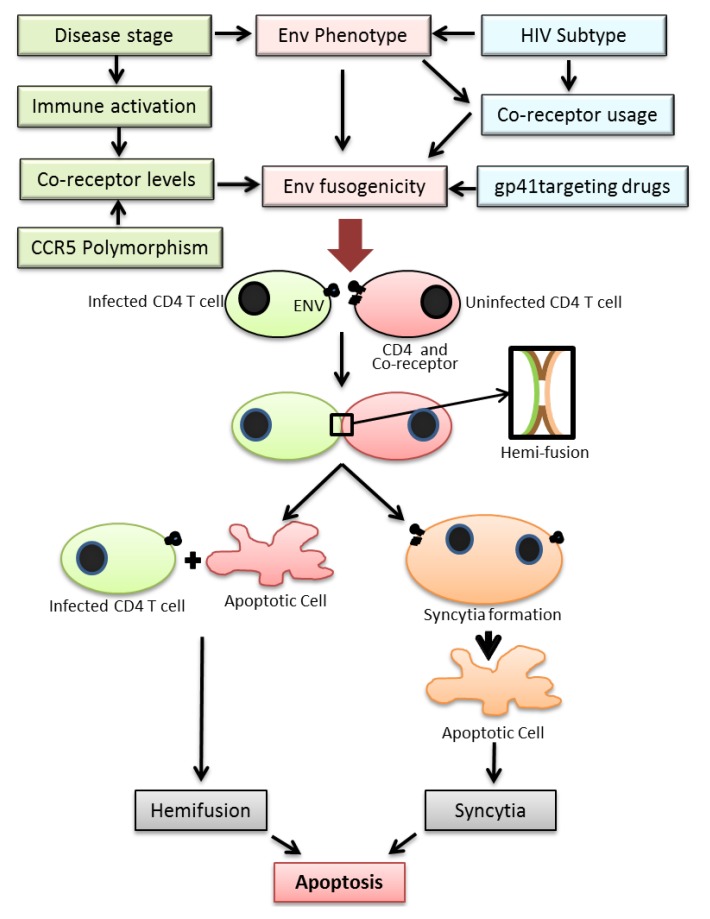
Figure depicting the various factors regulating HIV Env fusogencity leading to bystander apoptosis. **Top:** The fusogenic potential of the HIV Env glycoprotein is determined not only by the HIV subtype but also stage of disease, expression levels of HIV co-receptor in CD4+ T cells in infected individuals and the use of drugs targeting the viral envelope. A complex interplay of the above factors determines the Env fusogenic potential which in turn determines bystander CD4+ T cell death. **Bottom:** The process of T cell death in HIV infections is initiated by the interactions of Env glycoprotein in infected cells and receptor/co-receptor on neighboring bystander cells. This interaction either leads to full fusion between two interacting cells leading to syncytia formation or an abortive fusion leading to hemifusion. Both the processes eventually lead to cell death via apoptosis.

Two major mechanisms have been proposed for cell death induced by the HIV Env. These include (1) bystander apoptosis via interaction of HIV Env expressing cells with surface receptors CD4 and CXCR4/CCR5 leading to syncytia formation that eventually yield to apoptosis [14], (2) Partial mixing of the outer lipid membranes without complete fusion (hemifusion) upon interaction of Env expressing cells with surface receptors/co-receptors on neighboring cells (Figure 1) [14,15]. Recently a study by Doitsh *et al.* have shown that abortive infection by HIV can also lead to bystander apoptosis in *ex vivo* cultures of human tonsil tissue [31]. 

### 4.1. Apoptosis of Syncytia Formed via gp41 Mediated Fusion

Studies by Perfettini *et al.* showed that fusion of cells mediated by gp41 leads to syncytia formation that subsequently undergo apoptosis [32]. The process of syncytia formation is a well-documented phenomenon both *in vitro* and *in vivo* and is a hallmark of HIV infections in humans, monkey models and mouse models of HIV infections. Moreover, syncytia formation has been linked to HIV pathogenesis and progression to AIDS, with syncytia inducing (SI) phenotype viruses appearing later during the disease and associated with rapid CD4+ T cell decline [33]. 

The process of syncytia formation starts by fusion of two cellular membranes that lie in close proximity, followed by mixing of their cytoplasmic contents and eventually the nuclear membranes leading to abortive entry into mitosis [34]. These biophysical events are thought to initiate the apoptosis cascade in fused cells in the absence of mitosis. The molecular events that lead to this process are activation of Cdk1 (cyclin B dependent kinase 1), NFkB phosphorylation, activation of lamin, disassembly of nuclear envelope leading to karyogamy or mixing of nuclear material [8,35,36,37,38,39]. At this point syncytia also exhibit p53 phosphorylation [32,38] leading to transcription of pro-apoptotic genes like Bax and activation of the mitochondrial pathway for apoptosis characterized by activation of caspases [40,41]. P53 activity during syncytial driven apoptosis in turn is modulated by two different kinases namely the p38 MAPK (Mitogen Activated Protein Kinase) as well as mTOR (Mammalian Target Of Rapamycin) which show enrichment in karyogemic nuclei [42,43]. How precisely syncytium formation leads to the activation of p38 MAPK and mTOR is yet to be determined. The relevance of this phenomenon is not limited to *in vitro* studies but may also hold true in the case of HIV infected patients. The levels of Cyclin B have been found to be elevated in T cells from HIV infected patients [44,45]. Moreover, in patients showing improvement with HAART, mTOR and p53 activation correlated with viral loads [38,42]. Finally, syncytia with phosphorylated p38 and p53 have been seen in lymph node biopsies as well as brains from HIV infected patients undergoing neurodegeneration but not from HIV patients without neuronal symptoms [32,43]. In a recent study Murooka *et al.* [46] demonstrated the formation of small syncytia in lymph nodes of HIV-1 infected humanized mice as a result of HIV Env mediated fusion. These interactions resulted in reduced motility of HIV infected cells and interruption of T cell recirculation. Whether the loss of syncytia formed *in vivo* is a result of apoptosis or other mechanism like immune clearance or disintegration remains to be seen.

### 4.2. Hemifusion Induced Apoptosis

Another phenomenon associated with HIV Env mediated apoptosis is the process of hemifusion induced by gp41. Hemifusion is a process that involves transient interaction of cellular membranes in a manner that results in mixing of only the outer leaflets of the plasma membrane bilayers [47]. The HIV Env glycoprotein has been demonstrated to be capable of mediating hemifusion between virus infected and bystander cells [28,48,49,50,51]. In this regard, Blanco *et al*. showed that apoptosis induced by the Env glycoprotein required intimate cell to cell contact and binding to the co-receptor CXCR4 that could be reversed by addition of CXCR4 inhibitor AMD3100 [27]. Later, they used the gp41 inhibitor to demonstrate that the process of Env induced cell death required gp41 mediated transfer of lipids between two opposing membranes (hemifusion) without complete fusion or syncytium formation [28]. Other studies have shown that gp41 mediated fusion/hemifusion is critical for apoptosis induction and binding of gp120 alone cannot induce apoptosis signaling by HIV Env [28,29,50,52]. We and others found that HIV gp41 mediated hemifusion initiates an apoptotic cascade that involves caspase 3 activation but is independent of FAS or other cell surface death receptor signaling like Tumor Necrosis Factor (TNF) or TNF-related apoptosis-inducing ligand (TRAIL) [28,50]. Further studies using gp41 mutants with varying levels of fusion activity, including a hemifusion restricted mutant, showed that bystander apoptosis correlates with cell to cell hemifusion induction but not necessarily with virus infection or replication [29]. Studies by other groups using similar techniques have recently demonstrated that caveolin-1 a scaffold protein present in lipid rafts is capable of modulating HIV gp41 mediated hemifusion and consequently bystander apoptosis [49]. More recently, Cunyat *et al.* demonstrated that the use of varied kinds of effector cells in assays to determine bystander apoptosis mediated by HIV Env plays an important role in this process and may account for some of the differences seen between laboratories [53]. Overall, studies by us and others strongly suggest that hemifusion induced by HIV gp41 remains as one of the leading phenomenon associated with HIV-1 Env mediated bystander apoptosis [15,28,29,48,49,50,51]. 

## 5. Env Glycoprotein Phenotype and Bystander Apoptosis

The constant and rapid evolution of HIV Env within a patient has been the focus of extensive research. The variability in HIV Env is also a major limitation in the development of a successful vaccine against HIV. On the other hand the Env phenotypic variation may also play a significant role in HIV bystander apoptosis. Some of the phenotypic characteristics associated with HIV Env that determine virus pathogenesis are described below.

### 5.1. Env Fusogenicity

The data from *in vitro* studies regarding HIV Env mediated fusion and apoptosis is in accordance with findings *in vivo* from infected patients and animal models of HIV infection. The phenotype of Env glycoprotein has long been associated with HIV pathogenesis. In the SHIV model of HIV infection the fusogenic activity of Env correlates directly with CD4 loss [54,55]. Along similar lines, the fusogenic activity of Env glycoprotein has been indirectly associated with HIV pathogenesis in clinical studies where the presence of highly fusogenic Syncytia Inducing (SI) phenotype has been associated with poor prognosis in patients [33,56], increased pathogenesis [57] and CXCR4 [58] tropism of viruses. Studies by us and others have shown that gp41 mediated fusion/hemifusion is critical for apoptosis induction and binding of gp120 alone is insufficient for apoptosis induction [28,29,50]. Moreover, apoptosis induction by HIV Env is gp41 dependent and correlates with cell to cell fusion/hemifusion induction but not with virus infection or replication [28,29,50] both *in vitro* [50] and *in vivo* [59].

### 5.2. Co-Receptor Usage

HIV Env glycoprotein is a highly variable protein that constantly evolves within a patient throughout the disease. It is estimated that the genetic variation in the Env gene is the highest compared to any other HIV-1 genes. This evolution of Env has been associated with disease progression and enhanced pathogenesis of late stage viruses compared to early or chronic stage viruses [60]. This phenomenon in many cases correlates with the use of CXCR4 as a co-receptor by late stage viruses [33]. It has also been suggested that a switch from R5 tropic to X4 tropic virus precedes the rapid CD4 decline and AIDS development in many cases [57]. One of the underlying reasons for an accelerated decline of CD4 cells with X4 viruses has been linked to the expression of CXCR4 on virtually all CD4+ T cells while CCR5 expression is limited to CD4+ T cells localized in the gut [61]. This is consistent with the massive depletion of gut associated CD4+ T cells in primary infection with R5 viruses early in the disease [62]. However, studies indicate that in 50% of the patients there is no co-receptor switch and AIDS associated viruses are R5 tropic. In a study by Sterjovski *et al*., the authors demonstrated that the Env genes from viruses isolated at the late stages of HIV infection are more fusogenic than early stage viruses and in some cases this phenomenon maps to Asn 362 located near the CD4 binding site [63]. Increased affinity for CD4 or co-receptor has also been correlated with bystander apoptosis inducing activity of HIV-1 Env in-vitro by Holm *et al.* [64]. These findings again underscore the critical role played by Env phenotype in HIV pathogenesis. 

### 5.3. Disease Stage

Although the above findings support the notion that Env fusion is related to pathogenesis not only for X4 but also R5 viruses, there is limited information on whether bystander cell apoptosis by Env genes from different stages of the disease are different. However, analysis of late stage Envs by Wade *et al*. demonstrated that in fact these Envs are characterized by higher apoptosis induction [65]. Interestingly, these AIDS associated R5 viruses have been shown to be more fusogenic than early asymptomatic phase viruses [66]. Thus, the possibility that late stage Envs are more pathogenic as a result of higher bystander apoptosis irrespective of their co-receptor usage is clearly a possibility. 

### 5.4. HIV Subtype

The high level of variability between different subtypes of HIV that in many cases correlates with disease progression rate has also been reported. The evolution of HIV is markedly different in different subtypes. The most well characterized phenomenon is co-receptor usage evolution that seems to vary considerably between subtypes [67,68,69]. Recently it was demonstrated that in subtype C HIV infections the virus fails to evolve to CXCR4 usage during the later stages of the disease and most of the AIDS associated viruses in these patients utilize CCR5 [70]. This is markedly different from other subtypes where evolution of CXCR4 usage correlates with end stage viruses. In another recent study, Ng *et al*. demonstrated that disease progression in subtype CRF01_AE infected individuals is much faster than subtype B infected individuals [71]. Thus phenotypic variations of the HIV Env glycoprotein between different subtypes may also be relevant to bystander apoptosis and disease progression. Whether subtype differences are related to Env fusogenicity or coreceptor usage remains to be determined experimentally. 

## 6. Co-Receptor Expression Levels and Bystander Apoptosis

Coreceptor switching from early CCR5 usage to late CXCR4 usage in HIV infections is associated with rapid CD4 decline and AIDS development. The fact that CXCR4 is expressed on virtually all CD4+ T cells compared to CCR5, which is seen in only 5%–10% of CD4+ T cells, has been suggested as the explanation behind this phenomenon. However the fact remains that in a large proportion of patients, the co-receptor switch is not required and patients with exclusively R5 viruses progress to AIDS. The complexity of R5 virus infection is further accentuated by the differential levels of R5 expression among individuals. The classical CCR5Δ32 deletion mutation is well characterized for HIV resistance and long term non-progressors [72]. CCR5Δ32 homozygous individuals are resistant to HIV infection due to lack of CCR5 expression; [73,74] while CCR5Δ32 heterozygous individuals progress either relatively slowly to AIDS or remain as long term non-progressors [73,75] Besides this well characterized deletion mutation; a polymorphism in the CCR5 promoter region has also been associated with disease progression [76]. The association of promoter polymorphism to disease progression is not clear, although levels of CCR5 expression have been suggested as a mechanism [77]. 

As HIV Env mediated fusion is an interplay between Env fusion activity and receptor/co-receptor expression levels [78], it is natural to speculate that bystander apoptosis is also influenced by co-receptor levels. Studies have shown that PBMCs from CCR5Δ32+/− individuals express lower levels of CCR5 which correlates with infectivity of the virus [79]. In a study by Scoggins *et al*. [80], SCID-hu mice reconstituted with CCR5Δ32+/− thymus grafts were resistant to CCR5 virus mediated CD4 cell loss even in the presence of virus replication. In a recent study, Paiardini *et al*. [81] presented data demonstrating that reduced CCR5 expression in the CD4+ central memory T cell compartment in Sooty Mangabeys (SM) limits SIV infection and progression to AIDS. In the same study, CD4+ T cell activation did not result in CCR5 up regulation in SM thereby protecting cells against SIV infection and/or bystander apoptosis. The importance of CCR5 expression is further emphasized by the effect CCR5 expression levels (within physiological limits) have on Env mediated fusion and virus replication [82,83]. Platt *et al*. used HeLa cell lines with different levels of CCR5 expression to demonstrate that CCR5 expression levels can affect fusion mediated by different R5 isolates [82]. Whether increased surface expression of CCR5 accounts for the Env hemifusion mediated apoptosis phenotype in certain R5 viruses remains a valid hypothesis. One of the caveats of experiments with R5 Env has been the lack of a T cell line with physiological levels of CCR5. To address this issue directly we developed T cell lines with different levels of CCR5. When tested for bystander apoptosis induction in a coculture experiment with HIV Env expressing cells we found that the levels of CCR5 correlated with bystander apoptosis induction [84]. More importantly bystander apoptosis induction was independent of virus replication as the virus replicated in cells with lower levels of CCR5 with an absence of bystander apoptosis. These findings directly support the hypothesis that in CCR5Δ32 +/− individual’s virus replication may not be the cause of CD4+ T cell decline. Furthermore, the levels of CCR5 maybe an important contributing factor in HIV disease progression via directly influencing bystander apoptosis. 

## 7. Autophagy Mediated by HIV-1 Env

Autophagy like apoptosis is an essential cellular mechanism that maintains homeostasis in higher organisms. It is involved in protein degradation and recycling, maintenance of cellular organelles, cell growth and restriction of intracellular pathogens including viruses, bacteria and parasites. Autophagy is a defense mechanism used by the cell to restrict incoming pathogens. This may result in (1) elimination of the pathogen, (2) modulation of this process by the pathogen to restrict their elimination or (3) organisms taking advantage of the autophagosomes for their own replication [85,86]. The process of autophagy is regulated by a highly specific group of genes called the autophagy related genes (Atg) and the process is implicated in diseases like cancers and neurodegeneration. Understanding of the role of autophagy in viral infections is still in its infancy. HIV-1, for instance, is known to modulate autophagy in several different ways. In cells productively infected with the virus autophagy is inhibited [87,88,89] to favor virus survival which is manifested by lowered expression of autophagy specific proteins like Beclin 1 and lipidated form of microtuble-associated protein light chain 3 (LC3-II) [87,89]. Interestingly, the HIV protein Nef plays a major role in this process by binding to several proteins that regulate the autophagy process [90,91,92,93,94,95]. On the other hand, HIV infection also induces autophagy in uninfected CD4+ T cells but not in cells of the monocytic lineage that come in contact with Env expressing cells [96]. This process is dependent on interaction of HIV Env with surface receptors/co-receptors independent of CD4/CXCR4 signaling pathways but dependent on the fusion process [97]. Although the process of autophagy initiated in uninfected CD4 bystander cells requires HIV gp41 mediated fusion, the precise mechanism behind this phenomenon is not clear. Proteomic analysis and use of inhibitors suggest an accumulation of Reactive Oxygen Species (ROS) in autophagic bystander cells [98]. Moreover, although siRNAs or inhibitors of autophagy reduce apoptosis a direct correlation between autophagy and apoptosis is yet to be established. Interestingly, while it is well known that HIV infects cells of the monocytic lineage, these cells are less prone to undergo apoptosis via direct virus infection or bystander apoptosis mediated by the viral Env. While these cells are indeed susceptible to autophagy induced by chemicals, their resistance to HIV Env induced autophagy is an interesting phenomenon. Differences in the membrane architecture of T cells versus monocytic cells may be the reason for the resistance of the latter to both Env induced bystander apoptosis and Env induced autophagy. While the differences between apoptosis and autophagy in some cases maybe subtle the end result is cell death and both phenomenon are currently believed to contribute to CD4 loss in HIV-1 infection [99]. 

## 8. Targeting HIV gp41 Mediated Fusion Can Alter Bystander Apoptosis Inducing Function of Env

The importance of gp41 in mediating bystander apoptosis makes it an attractive target for therapy. Enfuvirtide was the first peptide inhibitor targeting gp41 induced fusion process that inhibits HIV entry in ways that parallel inhibition via neutralizing antibodies [100]. Targeting HIV gp41 via Enfuvirtide therapy still holds the caveat of emergence of resistance isolates, similar to other drugs that target miscellaneous viral proteins [101]. However, there seems to be an advantage associated with the development of resistance against Enfuvirtide which has been shown to affect HIV Env fusogenic properties [102]. The hypothesis that HIV gp41 fusion/hemifusion activity correlates with apoptosis induction suggests that drugs targeting gp41 function may alter HIV pathogenesis. In this context, it has been suggested that Enfuvirtide therapy may have beneficial effects by directly inhibiting gp41 mediated bystander cell death [103]. Recently Bonora *et al*. found that the addition of Enfuvirtide to HAART therapy regimen can result in greater and faster immunological recovery possibly via effects on bystander apoptosis [104]. Furthermore, the effect of Enfuvirtide on bystander cell death is not restricted to direct inhibition of gp41 function. In a clinical study, Aquaro *et al*. [105] showed that certain resistant viruses emerging during Enfuvirtide therapy are associated with CD4 increase in patients even after virological failure. These mutations are localized in the gp41 HR1 region and are known to affect gp41 fusion activity [102]. These findings were confirmed by Melby *et al*. [106] who reported that mutations at position V38 are associated with increase in CD4 recovery in Enfuvirtide treated patients after virological failure. Similar findings have recently been reported by Svicher *et al*. [107]. Our *in vitro* data suggest that the resistant mutants arising as a result of Enfuvirtide therapy have reduced cell to cell fusion capacity. This also parallels the reduced bystander apoptosis induction by these mutants while retaining virus infection and replication capacity [108]. In a more recent study, Cunyat *et al*. showed that the presence of V38A mutation in combination with N140I polymorphism is associated with reduced HIV mediated cytopathic effects [109]. These findings are in agreement with above mentioned clinical findings showing an increase in CD4 counts even after virological failure in Enfuvirtide treated patients [105,106,107]. Overall a reduction in virus pathogenesis via point mutations in gp41 supports the hypothesis that gp41 mediated fusion/hemifusion is critical for HIV pathogenesis. Furthermore, targeting Env mediated fusion by inhibitors of gp41 should inhibit both virus replication and Env-mediated bystander cell death and may select for resistant mutants that are less pathogenic. 

## 9. Immune Activation in HIV Disease Progression

Pathogenic HIV infections can be distinguished from non-pathogenic SIV infections by the presence of immune activation [110,111] seen in humans but not in natural infections in Sooty Mangabeys [112,113]. As HIV selectively targets CD4+ T helper cells of the immune system, it is not surprising that it induces immune dysfunction including immune activation. Whether HIV induced CD4+ T cell loss is also a consequence of immune activation remains to be determined. However, chronic immune activation remains a hallmark of pathogenic HIV infections and correlates with disease progression [110,114,115]. The activation of CD4+ T cells as determined by surface expression of activation markers like Ki67, HLA-DR, CD25 and CD38 [116,117] has been associated with HIV disease progression. In fact immune activation is a better predictor of disease progression than plasma viremia [118,119,120,121].

Although it is widely accepted that pathogenic HIV infections lead to chronic immune activation it is not clear what mediates this phenomenon. Immune activation has been shown to occur in isolated lymph node histocultures and requires active virus replication [122]. Interestingly, HIV infection of resting T cells most often results in latent infection and immune activation drives the virus into productive replication. Also immune activation leads to an up regulation of co-receptors, both CXCR4 and CCR5, that not only facilitate virus infection [123] but may also enhance Env mediated apoptosis. Studies in *ex vivo* human lymphoid tissue with HIV-1 show a unique pattern of T cell activation, characterized by CD25+/HLADR+ cells that facilitate virus replication [122]. Interestingly, the start of HAART therapy in patients also leads to decreased immune activation suggesting that the immune activation is virus dependent, although the mechanism is not clear [124]. Among several hypotheses proposed for immune activation, the role of gut leakage has been extensively studied. Brenchley *et al.* [125] demonstrated that early loss of CD4 cells in Gut Associated Lymphoid Tissue (GALT) resulted in gut leakage and was associated with increased lipopolysaccharide (LPS) levels in the blood causing immune activation. One hypothesis is that the apoptosis of intestinal epithelial cells causes breakdown of intestinal barrier resulting in microbial translocation that leads to immune activation. However, others have suggested that the microbial translocation may be a consequence rather than a cause of advanced HIV disease and AIDS [126]. Interestingly, immune activation paralleling human studies has recently been reported in humanized mouse model by Brainard *et al.* [127]. Hence a positive correlation between immune activation and CD4 loss clearly exists although it remains unknown whether HIV Env mediated bystander apoptosis drives the immune activation or it is a consequence of other mechanisms like gut leakage. 

## 10. Bystander Apoptosis in HIV Infected Individuals

HIV infections *in vivo* lead to accelerated CD4+ T cell death. This is seen not only in the infected but also the uninfected cell population. Moreover HIV infections also affect B cell and CD8 T cell function most likely due to lack of adequate cognate CD4 help that is essential for proper functioning of both T and B cell population. As a result HIV infections alter homeostasis of the entire immune system that may be at least in part due to apoptosis [128]. Various studies have tried to address the phenomenon behind accelerated apoptosis in T cells in HIV infected patients. Analysis of blood cells derived from HIV patients when cultured *in vitro* has revealed accelerated cell death in both infected as well as uninfected T cells [129]. Studies aimed at elucidating the mechanism behind this phenomenon have shown that PBMCs from HIV positive patients that show typical progression to AIDS have higher Caspase-3 and Caspase-9 activity with reduced Bcl2 levels [130] markers typically associated with apoptosis. This phenomenon is also associated with mitochondrial damage which correlates with reduced mitochondrial membrane potential in CD4+ T cells from HIV positive patients [131,132]. This observation is further complicated in acute HIV infections/typical progressors versus elite controllers/long term non-progressors that exhibit deviations from the above observations due to several known and unknown reasons. The TNF and TNF-R associated death pathway has also shown to be activated in HIV infected patients which makes cells of both the CD4 and CD8 lineage susceptible to apoptosis [133]. 

## 11. Bystander Apoptosis in Animal Models of HIV Infection

Answers to some of the fundamental questions regarding AIDS have been facilitated by the development of animal models of HIV infection. One of the most extensively used models is the SIVmac infection in Rhesus Macaques (RM) versus SIVsm infection in Sooty Mangabeys (SM) or SIVagm in African green Monkeys. While SIVsm infection in SM results in a nonpathogenic infection characterized by high levels of viremia in the absence of CD4 decline; infection of RM with SIVmac results in rapid loss of CD4 cells leading to immunodeficiency [5,134,135]. The differential pathogenesis in these models has been the focus of extensive research trying to understand the mechanism behind HIV pathogenesis. Interestingly, SIVmac infection in RM is characterized by a lack of bystander pathology and limited immune activation compared to SIVsm in SM [113]. A direct demonstration of differential apoptosis in the lymph nodes of SIVmac infected RM versus SIVagm infected AGMs was conducted by Cumont *et al.* [136]. In the same study the differential rate of disease progression in Indian RM versus Chinese RM infected with SIVmac also correlated with differential apoptosis in the two groups. Similar species specific differences in the innate immune response have also been documented by others to be responsible for the bystander apoptosis seen in these SIV models [137]. Furthermore in acute SIVmac infections in RM nonspecific immune activation has been suggested to be the cause of apoptosis in this model [138]. Other related models like the SHIV (SIV virus containing HIV Env, Rev and Nef genes) have also been used for studying HIV pathology in primates [139]. Interestingly in a SHIV model, the passage of SHIV89.6 in macaques resulted in a highly pathogenic SHIV89.6P variant. Further analysis of the molecular clone SHIVKB9 showed that the Env glycoprotein [140,141] and more specifically the membrane fusing activity of SHIVKB9 Env was associated with CD4 cytopathic effects *in vitro* as well as the pathogenesis *in vivo* [54,55,142]. 

The testing of HIV pathogenesis *in vivo* has also been greatly facilitated by the development of mouse model systems like the SCID-hu, hu-PBL-SCID and humanized mice systems [143]. In the humanized mouse model (Hu-HSC) reconstitution of CD34+ stem cells derived from the cord blood in either NOD/Lt-scidIL2rγ^−/^^−^ or the equivalent Rag^−/^^−^γc^−/^^−^ mice leads to development of a fairly representative human immune system. These human immune cells are susceptible to HIV infection by both CXCR4 utilizing and CCR5 utilizing strains and can support virus infection for several months [144]. Humanized mice provide a simple model to study HIV pathogenesis without the need for more expensive systems like SHIV or SIV infection of rhesus macaques while providing fairly similar pathology to human infections in terms of virus replication and CD4 loss [144,145,146]. What is uncertain is whether the mechanism of T cell loss in this model is the same as in human population. In fact, studies by Baenziger *et al.* [147] demonstrated a selective loss of CD4 cells and enhanced pathogenicity of X4 versus R5 viruses in the above mouse model. However, the mechanism behind this accelerated CD4 loss by X4 viruses compared to R5 viruses remains unclear. Although virus infection can be detected in different tissues in these animals, the loss of CD4 cells seems inconsistent with direct infection owing to the relatively few infected cells seen by histopathology. Syncytia inducing phenotype has also been associated with pathogenesis in SCID-hu mouse model suggesting a role for Env mediated fusion in pathogenesis in mouse model of HIV [148]. Recently, we have utilized the humanized mouse model of HIV infection to study the differential pathogenesis of a cell to cell fusion defective HIV Env mutant V38E versus Wild type. Interestingly we found that CD4+ T cell decline was minimal in mice when the viral Env was incapable of syncytia induction as in the case of V38E mutant. In the same study we found that bystander apoptosis was limited in V38E infected mice compared to Wild type which also correlated with CD4 decline [59]. Thus, findings from *in-vitro* studies, *in-vivo* from HIV infected patients, and an animal model of HIV infection indicate a role for HIV Env mediated fusion and bystander apoptosis in progression to AIDS.

## 12. Conclusion

Over the years an increasing amount of data has been accumulating regarding the role of bystander apoptosis induction in HIV infection and its role in disease progression. It has become evident that the process is not as simple as previously thought. A number of host and viral factors discussed in this review actually work in concert to regulate this phenomenon (Figure 1). While a lot more evidence has been gathering around the role of HIV Env and more interestingly the role of gp41 mediated hemifusion in HIV induced T cell loss, there are many questions that remain unanswered. For one, it is not known what biochemical or biophysical changes are happening at the hemifusion site that maybe responsible for initiation of apoptotic cascade. Secondly, it is unclear whether the phenomenon of Env mediated bystander apoptosis has relevance to disease progression in humans although animal models support this idea. Finally, it remains to be validated whether there are ways to attenuate the bystander apoptosis inducing activity of the virus by targeting the Env glycoprotein. Interestingly gp41 mutants commonly arising during Enfuvirtide therapy that have been shown to be associated with increased CD4 counts are less fusogenic in potential. Thus, the hypothesis that Env mutants with reduced fusion activity are less pathogenic and can be selected with the use of Enfuvirtide provides a novel concept in HIV treatment. Unlike conventional therapy where the objective is largely to eliminate virus replication, the concept behind targeting the HIV envelope is to establish whether HIV can be attenuated (in terms of bystander apoptosis induction) by mutations in the Env glycoprotein [149]. Analysis of HIV pathogenesis and CD4+ T cell decline via this new perspective will open new areas for anti-HIV therapy [149] aimed at finding novel means of virus attenuation by targeting the Env glycoprotein.
